# Assessment of diversity-based approaches used by American Universities to increase recruitment and retention of biomedical sciences research faculty members: A scoping review protocol

**DOI:** 10.1371/journal.pone.0276089

**Published:** 2023-06-22

**Authors:** Britta Petersen, Sherli Koshy-Chenthittayil, Megan DeArmond, Leslie A. Caromile

**Affiliations:** 1 Center for Molecular Oncology, UConn Health, Farmington, Connecticut, United States of America; 2 Office of Institutional Effectiveness, Touro University Nevada, Henderson, Nevada, United States of America; 3 Jay Sexter Library, Touro University Nevada, Henderson, Nevada, United States of America; 4 Center for Vascular Biology, UConn Health, Farmington, Connecticut, United States of America; Oxford University Hospitals NHS Foundation Trust, UNITED KINGDOM

## Abstract

Diversity enriches the educational experience by improving intellectual engagement, self-motivation, citizenship, cultural engagement, and academic skills like critical thinking, problem-solving, and writing for students of all races. Faculty role models from similar backgrounds are essential for students from traditionally underrepresented groups as it sends a powerful message of support, belonging, and the confidence to pursue higher education. However, in the biomedical sciences, the percentage of historically underrepresented tenure-track faculty is far lower than that of their white colleagues. For this to change, a strong strategic plan and commitment from the university are imperative. This scoping review will assess the size and scope of available peer-reviewed research literature on diversity programs that aim to increase the recruitment and retention of biomedical sciences research faculty and are implemented and evaluated at American Universities. The information provided in this scoping review will help universities identify novel, successful diversity-based approaches for recruiting and retaining biomedical science faculty that might suit their own unique academic and geographic needs and be incorporated into their diversity initiatives and policies. The review follows the Population-Concept-Context methodology for Joanna Briggs Institution Scoping Reviews. Relevant peer-reviewed studies published in English between June 1, 2012, to June 1, 2022, will be identified from the following electronic databases; MEDLINE (PubMed), Scopus (Elsevier), EMBASE (Elsevier), CINAHL (EBSCO), and ERIC (EBSCO). The search strings using the key variables “biomedical research faculty,” “recruitment/retention,” “diversity/ minority/ underrepresented, and “mentoring” will be conducted using Boolean logic. Two independent reviewers will conduct all title and abstract screening, followed by a full article screening and data extraction. Due to the possible heterogeneity of the studies, we hope to use either a narrative analysis and/or descriptive figures/tables to depict the results.

## Introduction

The need for more innovative approaches to diversify biomedical sciences research faculty is evident in the changing demographics of the student body at American universities. While the national percentage of historically underrepresented college students (undergraduate and graduate students combined) has risen to nearly 50%, the rate of historically underrepresented faculty (Blacks or African Americans, Hispanics or Latinos, American Indians or Alaska Natives, Native Hawaiians, and other Pacific Islanders), remains below 30% [1–5]. The percentage of historically underrepresented tenure-track faculty remains even lower at 22% [6]. These numbers represent the racial and ethnic categories and do not include a breakdown of other underrepresented groups, i.e., LGBTQ+, veterans, and gender minorities. The benefits of a more diverse faculty extend to all students [6]. For students from traditionally underrepresented groups, engaging faculty role models from similar backgrounds is essential, as it sends a powerful message of support, belonging, and the confidence to pursue higher education [4]. As for students from majority backgrounds, the benefit lies in the experience of broader pedagogical perspectives [7] and the opportunity to counter stereotypes and reduce bias [8]. Therefore, it is imperative that universities not only adopt a campus-wide commitment to faculty diversity but also adopt policies and initiatives that support that commitment if they want to have a diverse student body.

The academy has been discussing strategies to improve diversity and equity in the biomedical sciences for decades, but progress has been incremental and slow. There is no “one-size fits all” solution. Different academic institutions and units within those institutions often need different approaches [9]. For example, smaller universities, primarily undergraduate institutions and community colleges within the United States, in large, have fewer funding sources than larger R1 universities and would not be able to implement diversity strategies that require a large financial investment. Additionally, geographic location, population of students being served as well as urban or rural setting can styme a “general” diversity initiative. Furthermore, the current definition of diversity and the focus on diversity initiatives has evolved dramatically since the early 2000s. For example, while gender diversity in senior-level academic positions remains an issue, the inclusion of ethnic and cultural diversity, including people with disabilities, in the workplace has increased. Additionally, in the early 2000’s, efforts focused on increasing the recruitment and participation of “racial and ethnic minorities”. However, in 2022, the word “minority” has become outdated, inaccurate, and potentially harmful. Finally, one of the largest changes can be seen within the LGBTQ+ community and the definitions of diverse sexual identities and gender expression.

Unfortunately, peer-reviewed, published research literature on the topic of recruitment and retention of diverse biomedical science research faculty is lacking. This makes it challenging to research and eventually create successful, diversity-centered policies that are appropriate for their needs. Where does one start? Here we present a protocol for a scoping review [10] where we will perform a preliminary assessment of the size and scope of available peer-reviewed research literature on diversity programs that have been implemented and evaluated at American Universities aimed to increase the recruitment and retention of biomedical sciences research faculty. We define diversity-based approaches as any strategies adopted to increase the presence of historically underrepresented populations in a particular space. In this review, the following fields and professional groups will be included: biological science disciplines (cell biology, immunology, molecular biology, pharmaceutical sciences, genetics, neuroscience, biomedical engineering), nursing, occupational therapy, physical therapy, and medical science. We will only evaluate strategies for research faculty within these fields and groups. Additionally, there are many federally national programs aimed at increasing diversity in health related research (e.g., The Summer Institute Program to Increase Diversity (SIPID)) [11]. The SIPID mentoring program continued as PRIDE (Program to Increase Diversity among Individuals Engaged in health-related research) [12], which was established before 2012. However, the impact of those programs was not reported until after 2012. Therefore, because of the changes in the definition of diversity over the past 20 years, we will only include literature from 2012 to the present in our search [13, 14]. Therefore, the aim of this scoping review is to provide curated information to help universities identify novel, successful diversity-based approaches for recruiting and retaining biomedical science faculty that might suit their unique academic and geographic needs, which can be incorporated into their diversity initiatives and policies.

## Methods

The stages of the review include [15]: (i) identifying the research questions; (ii) identifying the relevant studies; (iii) study selection; (iv) charting the data; (v) collating, summarizing, and reporting the results; and (vi) consultation.

### (i) Identifying the research question

Based on the Population, Concept, Context (PCC) [15] framework put forward by JBI, the research question for this scoping review is “What are the diversity-based approaches used by American Universities to increase recruitment and retention of biomedical sciences research faculty members?”. The PCC with regards to our review is shown in Table 1.

**Table 1:** Population-Concept-Context methodology.

Population	Biomedical Sciences Research Faculty Members
Concept	Diversity-based approaches
Context	American Universities

### (ii) Identifying the relevant studies

For the initial search, we will consult with a medical reference librarian from UConn Health and Touro University for subsequent searches. Preliminary searches will be conducted in MEDLINE (PubMed), Embase, and Scopus to identify articles on the topic, review existing strategies, and search for published articles. The results will be exported to Endnote for title and abstract screening. The initial search used MeSH terms to identify keywords and controlled vocabulary using the MEDLINE (PubMed) platform and will be further adapted to other databases. (S1 Table in S1 File).

For the full scoping review, articles outside of the publication date range will be excluded during title and abstract screening. Papers not published in English that meet inclusion criteria in the title and abstract screening will be reviewed for usable data. Due to restrictions in funding and author language limitations, papers not in English will be excluded at the full text screening stage. The search strategy will be adapted for each included database. The databases to be searched include MEDLINE (PubMed), Scopus (Elsevier), EMBASE (Elsevier), CINAHL (EBSCO), ERIC (EBSCO).

### (iii) Selection of studies

**Fig 1:**
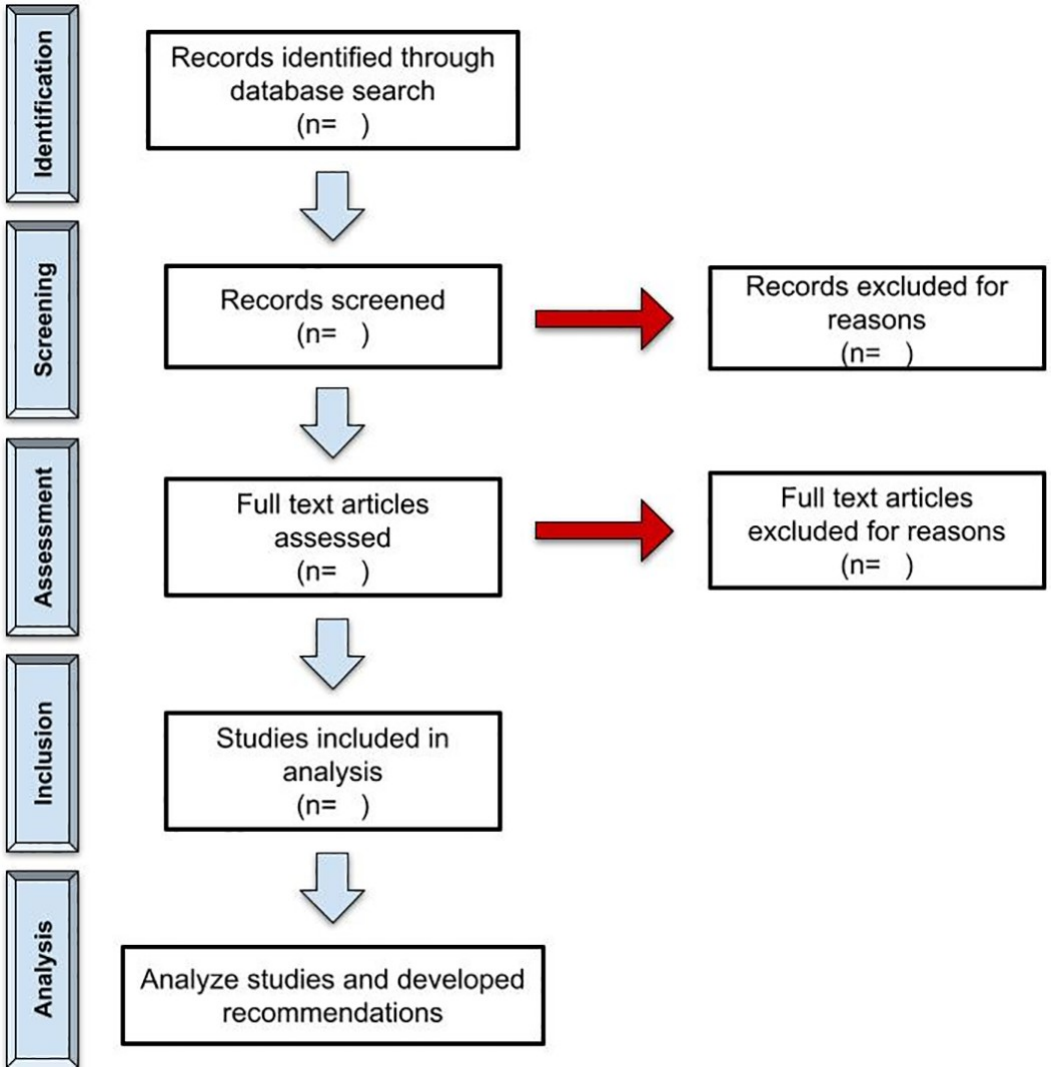
Selection of studies according to PRISMA-ScR protocol.

Two independent reviewers will screen the records from the databases utilized using the title and abstract. The reviewers will then assess the full text articles and will decide based on the inclusion/exclusion criteria given below. Any disagreements will be discussed between the two reviewers until consensus is reached, or a third reviewer will be a tiebreaker. Reasons for exclusion will be noted, and the process of study selection will be documented in a flow chart (Fig 1), according to Preferred Reporting Items for Systematic reviews and Meta-Analyses extension for Scoping Reviews (PRISMA-ScR) [16].

In this review, we will be evaluating diversity-based approaches exclusively performed with US biomedical research institutions. Diversity is defined differently in various countries due to historical context. For example, due to the caste system of India, the diversity strategies used in Indian universities will need to address the attitudes towards the historically oppressed castes. In the US, since there is limited acknowledgement of the caste system, the caste-based strategies would be ineffective. Additionally, the historical experiences of African Americans and Native Americans here in the US, cannot be rendered outside of the US. Hence, we believe the cultural context of diversity-based strategies is important and there may be a translational issue when we consider approaches from outside of the US.

As others have reported [17–19], and after preliminary searching of gray literature and university websites, implementation and outcome measures were often not reported. Most of the diversity-related policies on websites do not cite nor share their data sources or methodology hence making it more difficult to analyze the quality of the work. The websites often lacked detailed information on how the policies were implemented as well as endpoints. Another point to note is that the heterogeneity, lack of peer-review and a deficiency of abstracts for gray literature make it harder to screen for inclusion/exclusion. Hence for this review, gray literature will be excluded.

Since we are excluding gray literature, we are modifying the existing Mixed Methods Appraisal Tool (2018) [20] for our included studies. The quality assessment questions are: Are the different components of the mixed-methods study effectively integrated to answer the research question? Are measurements appropriate regarding both the outcome and intervention? Are the data collection methods adequate to address the research question?

#### Inclusion criteria for this study

- Articles published from June 1, 2012, to June 1, 2022.
- An original intervention or observation with outcomes or evaluation data on retention, recruitment, or prevalence of historically underrepresented biomedical sciences research faculty.
- Indexed journals published in MEDLINE (PubMed), Scopus (Elsevier), EMBASE (Elsevier), CINAHL (EBSCO), ERIC (EBSCO).
- Articles that focused on historically underrepresented biomedical sciences research faculty recruitment, retention, and mentoring.
- Qualitative studies with pre- and post-intervention data.
- Written in English.

#### Exclusion criteria for this study

- Papers that were narrative reviews, expert opinion, editorials, or letters to the editor.
- Papers that were published before June 1, 2012.
- Papers that did not include any data with their description.
- Books or book chapters.
- Websites that provide unpublished, non-peer-reviewed internal statistics.

### (iv) Charting the data

We will extract the following information from the selected studies: authors, title, year of publication, database used, diversity-based approach used to increase recruitment and retention of biomedical sciences research faculty, number of participants in the study, and the results of the evaluation of the approach. We will contact the authors of the included papers if pertinent information is missing or unclear. To ensure consistency, the data will be extracted by one reviewer and validated by another. The data-charting will be updated iteratively based on the studies found.

### (v) Collating, summarizing, and reporting the results

Since this is a scoping review, the results of the data extraction will provide an overview of the different approaches adopted by American universities to increase the recruitment and retention of diverse biomedical sciences research faculty. Due to the possible heterogeneity of the studies, we hope to use either a narrative analysis and/or descriptive figures/tables to depict the results. The final manuscript will follow the PRISMA-ScR format extension [16].

### (vi) Consultation

The results will be disseminated to the relevant stakeholders, including biomedical sciences research faculty members, university diversity, equity and inclusion councils, policy-decision makers, and deans. This will be done using paper publication, and presentations at national conferences such as ASCB (American Society for Cell Biology), ASBMR (American Society for Bone and Mineral Research), SACNAS (Society for the Advancement of Chicanos and Native Americans in Science) and sharing with our institutions’ DEI councils. The link to the open-source paper will be publicly available through our institutions’ DEI websites.

## Discussion

College faculty have become more racially and ethnically diverse but remain far less so than students [21]. When assessing diversity in the biomedical sciences, both the National Institutes of Health and the National Science Foundation have showed the following racial and ethnic groups to be historically underrepresented: Blacks or African Americans, Hispanics or Latinos, American Indians or Alaska Natives, Native Hawaiians, and other Pacific Islanders. Additionally, women, LGBTQ, veterans, and gender minorities have been shown to be underrepresented in doctorate-granting research institutions at senior research faculty levels in most biomedical-relevant disciplines [22].

Although many diversity-based initiatives that aim to increase the aforementioned groups have been locally successful, the data gathered, and their outcomes are rarely published. This makes it hard to implement these policies. This scoping review will provide information on peer-reviewed, diversity-based approaches used by American Universities to increase the recruitment and retention of underrepresented biomedical sciences research faculty members. The strengths of the scoping review will include original, peer-reviewed interventions or observations with outcomes or evaluation data on retention, recruitment, or prevalence of historically underrepresented biomedical sciences research faculty; information focusing on historically underrepresented biomedical sciences research faculty recruitment, retention, and mentoring; and qualitative studies with pre- and post-intervention data. Having all this critically appraised data in one place will be helpful for American biomedical science research institutions to research and implement their own policies to increase the recruitment and retention of underrepresented biomedical sciences research faculty members based on their needs. However, there are several potential limitations of the scoping review, including that biomedical sciences research faculty underrepresentation can vary from setting to setting and, therefore, different approaches may be needed [9]. In conclusion, we believe that despite these limitations, our future scoping review will help universities identify novel, successful diversity-based approaches for recruiting and retaining biomedical science faculty that might be applicable to their university’s unique needs instead of using a general approach.

## Supporting information

S1 File (PDF)

## References

[1] Brown S. Diversifying your campus: Key insights and models for change. United States: Chronicle of Higher Education. 2021:9–14.

[2] Espinosa LL, Turk JM, Taylor M, Chessman HM. Race and ethnicity in higher education: A status report. 2019.

[3] Finkelstein MJ, Conley VM, Schuster JH. The faculty factor: Reassessing the American academy in a turbulent era: JHU Press; 2016.

[4] Griffin KA. Institutional barriers, strategies, and benefits to increasing the representation of women and men of color in the professoriate: looking beyond the pipeline. Higher Education: Handbook of Theory and Research: Volume 35. 2019:1–73.

[5] Shin JEL, Levy SR, London B. Effects of role model exposure on STEM and non-STEM student engagement. Journal of Applied Social Psychology. 2016;46(7):410–27.

[6] Snyder TD, De Brey C, Dillow SA. Digest of Education Statistics 2014, NCES 2016–006. National Center for Education Statistics. 2016.

[7] Umbach PD. The contribution of faculty of color to undergraduate education. Research in higher education. 2006;47(3):317–45.

[8] Gocłowska MA, Crisp RJ. On counter-stereotypes and creative cognition: When interventions for reducing prejudice can boost divergent thinking. Thinking skills and creativity. 2013;8:72–9.

[9] Asai DJ. Race Matters. Cell. 2020;181(4):754–7. Epub 2020/05/16. doi: 10.1016/j.cell.2020.03.044 . 32413295

[10] Munn Z, Peters MD, Stern C, Tufanaru C, McArthur A, Aromataris E. Systematic review or scoping review? Guidance for authors when choosing between a systematic or scoping review approach. BMC medical research methodology. 2018;18:1–7. 30453902 10.1186/s12874-018-0611-x PMC6245623

[11] https://grants.nih.gov/grants/guide/rfa-files/rfa-hl-07-012.html: National Heart, Lung, and Blood Institute. The Summer Institute Program to Increase Diversity (SIPID).

[12] https://www.nhlbi.nih.gov/grants-and-training/training-and-career-development/programs-increase-diversity-among-individuals: National Heart, Lung, and Blood Institute. PRIDE (Program to Increase Diversity among individuals Engaged in health-related research.

[13] https://www.vsource.io/blog/evolution-of-diversity-in-the-workplace [cited 2023].

[14] https://www.pewresearch.org [cited 2023].

[15] Peters MD, Godfrey C, McInerney P, Munn Z, Tricco AC, Khalil H. Chapter 11: scoping reviews (2020 version). JBI manual for evidence synthesis, JBI. 2020;2020.

[16] Tricco AC, Lillie E, Zarin W, O’Brien KK, Colquhoun H, Levac D, et al. PRISMA Extension for Scoping Reviews (PRISMA-ScR): Checklist and Explanation. Ann Intern Med. 2018;169(7):467–73. Epub 2018/09/05. doi: 10.7326/M18-0850 . 30178033

[17] Benzies KM, Premji S, Hayden KA, Serrett K. State-of-the-evidence reviews: advantages and challenges of including grey literature. Worldviews on Evidence-Based Nursing. 2006;3(2):55–61. doi: 10.1111/j.1741-6787.2006.00051.x 17040510

[18] Mahood Q, Van Eerd D, Irvin E. Searching for grey literature for systematic reviews: challenges and benefits. Research synthesis methods. 2014;5(3):221–34. doi: 10.1002/jrsm.1106 26052848

[19] Turner AM, Liddy ED, Bradley J, Wheatley JA. Modeling public health interventions for improved access to the gray literature. Journal of the Medical Library Association. 2005;93(4):487. 16239945 PMC1250325

[20] Hong QN, Fàbregues S, Bartlett G, Boardman F, Cargo M, Dagenais P, et al. The Mixed Methods Appraisal Tool (MMAT) version 2018 for information professionals and researchers. Education for information. 2018;34(4):285–91.

[21] Davis LF, Richard. College faculty have become more racially and ethnically diverse, but remain far less so than students: Pew Research Center; 2019. https://www.pewresearch.org/fact-tank/2019/07/31/us-college-faculty-student-diversity/.

[22] Populations Underrepresented in the Extramural Scientific Workforce Web: National Institutes of Health; 2022. https://diversity.nih.gov/about-us/population-underrepresented.

